# Mechanotransduction via the coordinated actions of integrins, PI3K signaling and Connexin hemichannels

**DOI:** 10.1038/s41413-020-00126-w

**Published:** 2021-02-02

**Authors:** Manuel A. Riquelme, Sumin Gu, Rui Hua, Jean X. Jiang

**Affiliations:** Department of Biochemistry and Structural Biology, University of Texas Health Science Center, San Antonio, TX 78229-3900 USA

**Keywords:** Bone quality and biomechanics, Bone

## Abstract

Mechanical loading opens connexin 43 (Cx43) hemichannels (HCs), leading to the release of bone anabolic molecules, such as prostaglandins, from mechanosensitive osteocytes, which is essential for bone formation and remodeling. However, the mechanotransduction mechanism that activates HCs remains elusive. Here, we report a unique pathway by which mechanical signals are effectively transferred between integrin molecules located in different regions of the cell, resulting in HC activation. Both integrin α5 and αV were activated upon mechanical stimulation via either fluid dropping or flow shear stress (FSS). Inhibition of integrin αV activation or ablation of integrin α5 prevented HC opening on the cell body when dendrites were mechanically stimulated, suggesting mechanical transmission from the dendritic integrin αV to α5 in the cell body during HC activation. In addition, HC function was compromised in vivo, as determined by utilizing an antibody blocking αV activation and α5-deficient osteocyte-specific knockout mice. Furthermore, inhibition of integrin αV activation, but not that of α5, attenuated activation of the phosphoinositide 3-kinase (PI3K)-protein kinase B (AKT) signaling pathway upon mechanical loading, and the inhibition of PI3K/AKT activation blocked integrin α5 activation and HC opening. Moreover, HC opening was blocked only by an anti-integrin αV antibody at low but not high FSS levels, suggesting that dendritic αV is a more sensitive mechanosensor than α5 for activating HCs. Together, these results reveal a new molecular mechanism of mechanotransduction involving the coordinated actions of integrins and PI3K/AKT in osteocytic dendritic processes and cell bodies that leads to HC opening and the release of key bone anabolic factors.

## Introduction

Bone continuously undergoes remodeling, which helps to maintain the proper structure and organization of the tissue. Mechanical loading, induced by healthy physical activity, promotes bone formation, and remodeling in association with enhancement of bone mass and strength.^1^ Osteocytes, the most abundant bone cell type, are thought to be the most likely mechanosensory cells in bone. Osteocytes were recently suggested to be the main factor regulating bone remodeling by orchestrating the functions of other bone cells as well as the remodeling of the bone matrix and are a potential therapeutic target for the treatment of osteoporosis.^2–4^ Osteocytes are embedded inside the bone mineral matrix, and the long dendritic processes of osteocytes form a network among neighboring osteocytes and cells on the bone surface.^5^ Osteocyte cell bodies and processes are surrounded by a fluid-filled space, creating an extensive lacunae-canalicular network.^6,7^ Interstitial fluid flow driven by extravascular pressure is thought to be a major form of mechanical stimulation for osteocytes.^8–11^

The small molecules generated by mechanical loading are likely transmitted between cells through gap junctions and between the cell and the extracellular matrix through hemichannels (HCs), which constitute half of all gap junction channels.^12^ HCs, formed by hexameric connexin molecules,^13^ have been demonstrated to be active in osteocytes in response to mechanical stress and are associated with the release of physiologically relevant anabolic molecules, such as prostaglandin E2 (PGE_2_), to the external environment.^14,15^ Prostaglandins released by bone cells are suggested to be skeletal anabolic agents, as they can increase bone mass in animals.^16–18^ As shown in our earlier study, HC activity is adaptively regulated by the magnitude and duration of flow shear stress (FSS).^19^ Our recent study also suggested that impairment of osteocytic HCs has a negative impact on cortical bone structure, strength, and osteocyte viability.^20^

Using a transwell filter system to separate cell bodies from dendritic processes, we demonstrated that dendritic processes sense mechanical stimulation and transmit signals to the cell body to open HCs.^21^ We further found that integrin α5β1 interacts directly with Cx43 and that this interaction is important for the opening of HCs on the cell body in response to mechanical loading.^22^ Mechanical stimulation facilitates the opening of Cx43 HCs, likely through PI3K activation.^23^ In addition, morphological and functional studies suggest that dendritic processes serve as osteocytic mechanosensory sites;^21,24,25^ integrins associated with other extracellular components serve as “tethering elements,” which connect the processes with the canalicular wall and amplify mechanical signals.^8,26^ One study showed that integrin αVβ3, located at the dendritic process, is responsible for the mechanosensory responses of osteocytes.^27^ Nonetheless, it remains unclear how mechanical signals are transmitted from the extended, long dendritic process to the cell body to open Cx43 HCs.

In this study, we unveil a new intracellular mechanotransduction pathway in mechanically sensitive osteocytes via which integrin αVβ3 at dendritic processes senses shear stress, transmits the signal to the cell body by activating intracellular PI3K-AKT signaling, and activates α5β1. This mechanotransduction leads to the opening of Cx43 HCs, which play an essential role in mediating the anabolic function of mechanical loading on bone.

## Results

### Activation of integrin αVβ3 at dendritic processes opens HCs on the osteocyte cell body through PI3K-AKT signaling

**Fig. 1:**
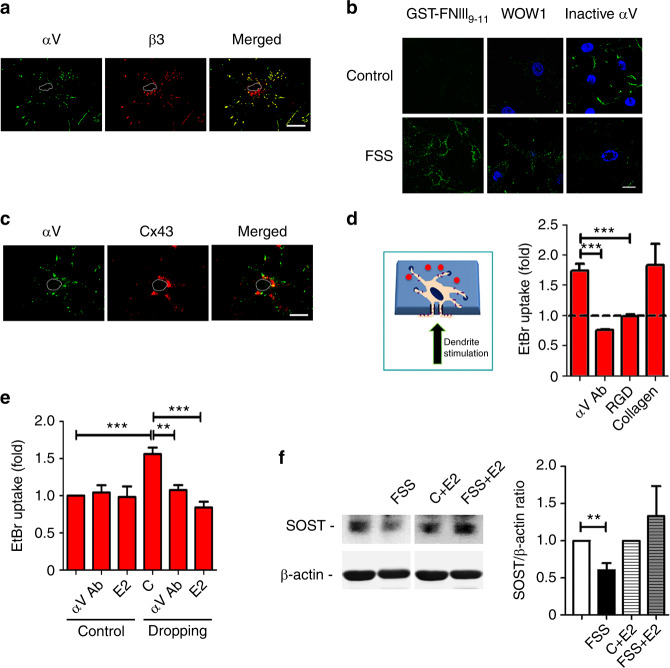
Integrin αVβ3 in osteocytes plays an important role in transmitting mechanical stimulation from dendrites to HCs located on the cell body. **a** Integrin αV colocalizes with β3 at the dendritic processes of osteocytes. MLO-Y4 cells were coimmunoprecipitated with an anti-integrin αV inhibitory antibody on the cell surface and with β3 antibodies in permeable cells, followed by incubation with FITC- and rhodamine-conjugated secondary antibodies, respectively. Bar, 10 µm. **b** FSS activates integrin αVβ3. MLO-Y4 cells were subjected to FSS at 8 dynes per cm^2^ for 30 min or not treated with FSS and then incubated with the GST-FNIII_9-11_ fragment, immunolabeled with a WOW1 antibody or incubated with an antibody targeting inactive integrin αV, followed by incubation with a FITC-conjugated corresponding secondary antibody. Bar, 10 µm. **c** Cell surface-expressed integrin αV does not localize with Cx43 in osteocytes. MLO-Y4 cells were coimmunoprecipitated with anti-integrin αV inhibitory and Cx43 antibodies in permeabilized cells, followed by incubation with FITC- and rhodamine-conjugated secondary antibodies, respectively. Bar, 10 µm. **d** Activation of integrin αV by mechanical loading in dendritic processes opens HCs on MLO-Y4 osteocyte cell bodies. MLO-Y4 cells cultured on transwell filter inserts were pretreated with an αV inhibitory antibody (αV Ab), 500 μmol·L^−1^ RGD peptide, or 2.5 μg·mL^−1^ collagen for 15 min. The cells were mechanically stimulated by dropping solution on the dendritic processes (bottom) side of the transwell filter (left panel), after which EtBr was added, and its uptake was quantified (right panel). The basal level of the dye uptake is indicated by the dashed line. The data are presented as the mean ± SEM. *n* = 3. ***, *P* < 0.001. **e** Activation of integrin αV by mechanical loading via liquid dropping opened HCs in IDG-SW3 osteocytes. Differentiated IDG-SW3 osteocytes were pretreated with an αV inhibitory antibody (αV Ab) or a Cx43 (E2) antibody for 15 min. The cells were mechanically stimulated by dropping solution, and EtBr uptake was quantified. The data are presented as the mean ± SEM. *n* = 3. *, *P* < 0.05; ***, *P* < 0.001. **f** HCs activated by FSS inhibited SOST expression. IDG-SW3 cells were treated with FSS for 2 h in the absence or presence of a Cx43(E2) antibody. Four hours after FSS, cell lysates were prepared and immunoblotted with an anti-SOST or anti-β-actin antibody (left panel), and band intensity was quantified (right panel). The data are presented as the mean ± SEM. **, *P* < 0.01. *n* = 3

Integrin αVβ3 has been implicated as part of a “tethering element” connecting the dendrites of osteocytes to the canaliculi wall.^8,25,26,28^ Immunofluorescence labeling with antibodies specific for integrin αV or β3 showed that these integrin subunits are located at osteocyte dendritic processes (Fig. 1a). These two integrin subunits are also colocalized, suggesting that they form αVβ3 heterodimers. We previously reported that FSS activates integrin α5β1 on the osteocyte cell body and that this activation and direct interaction between α5β1 and Cx43 opens HCs.^22^ MLO-Y4 osteocytic cells were subjected to FSS, and untreated cells were used as controls. Activation of integrin α5β1 was assessed based on increased binding to a GST-FNIII_9-11_ fragment;^22,29^ activation of αVβ3 was evaluated by either increased binding to WOW1 Fab^30^ or decreased binding with an inhibitory antibody targeting inactive integrin αV^31^ (Fig. 1b). Similar to integrin α5β1, FSS activated αVβ3. However, unlike integrin α5β1, integrin αV did not colocalize with Cx43, which predominately localized on the cell body, as determined by immunofluorescence (Fig. 1c). To evaluate whether αVβ3 is the mechanosensor responsible for HC opening in the cell body, we applied mechanical stress by dropping a liquid on the side of a transwell penetrated by dendritic processes (Fig. 1d, left panel). Mechanical loading by dropping on dendritic processes in transwell filters increased the uptake of ethidium bromide (EtBr) dye, an indicator of HC opening, on the cell body. The HC opening in the cell body was completely attenuated by pretreatment with an integrin αV inhibitory antibody or an integrin-binding RGD peptide on dendritic processes (Fig. 1d, right panel); this inhibition was not observed with collagen, which cannot bind to αVβ3.

To further define the role of αVβ3 in Cx43 HC activation, we conducted similar studies in differentiated IDG-SW3 osteocytes. Culturing the cells for 9 days in differentiation media resulted in the formation of osteocytes, manifested as increases in the GFP signal^32^ and Cx43 expression (Fig. S1A). Similar to the phenomenon in MLO-Y4 cells, mechanical loading via liquid dropping opened HCs, which was significantly inhibited by the Cx43(E2) antibody (Fig. S1B). Mechanical loading on dendritic processes opened HCs on the cell body, and this opening was significantly inhibited by the αV activation blocking antibody and the Cx43(E2) antibody (Fig. 1e). Sclerostin (SOST), a Wnt signaling inhibitor, is one of the major proteins expressed by osteocytes^33^ and is thus a negative regulator of bone formation. FSS significantly reduced SOST expression in IDG-SW3 cells, and this inhibition was ablated by the inhibition of HC opening by the Cx43(E2) antibody (Fig. 1f). These data suggest that Cx43 HCs mediate the anabolic function of osteocytes in response to mechanical loading.

**Fig. 2:**
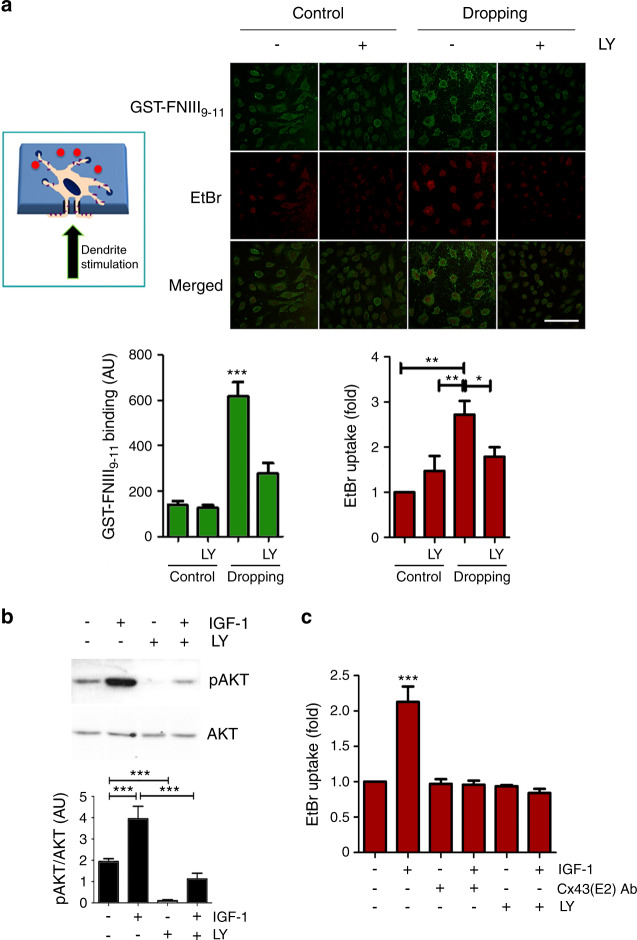
Activation of PI3K by mechanical loading is essential for HC opening. **a** Inhibition of PI3K signaling prevents the activation of α5β1 and the opening of HCs by mechanical loading. MLO-Y4 cells preincubated with or without 10 μmol·L^−1^ LY294002 (LY) for 15 min were subjected to mechanical loading by liquid dropping with 50 μmol·L^−1^ EtBr solution to the dendritic process side of the transwell filter (upper left panel). The cell body side of the transwell filter was then incubated with the GST-FNIII_9-11_ fragment. The confocal fluorescence images were taken; green fluorescence indicates binding of the GST-FNIII_9-11_ fragment, and red fluorescence indicates the uptake of EtBr (upper right panel). The extents of α5 activation (lower left panel) and HC opening (lower right panel) were determined based on GST-FNIII_9-11_ binding and EtBr uptake, respectively. Bar, 100 µm. Cells under dropping versus all other treatments, *, *P* < 0.05; **, *P* < 0.01; ***, *P* < 0.001. **b** Activation of PI3K-AKT by IGF-1 in osteocytes. MLO-Y4 cells were pretreated with or without LY and then incubated with 5 ng·mL^−1^ IGF-1 for 30 min. Cell lysates were immunoblotted with an anti-phospho-AKT (Ser473) (pAKT) or total AKT antibody. The band intensity and the ratio of phosphorylated to total AKT were quantified (lower panel). **c** The opening of HCs requires PI3K activation. MLO-Y4 cells were pretreated with or without LY or a Cx43(E2) antibody for 15 min and then incubated with 5 ng·mL^−1^ IGF-1. HC activity was then measured by EtBr dye uptake. All data are presented as the mean ± SEM. *n* = 3. Cells treated with only IGF-1 versus all other treatments and control, ***, *P* < 0.001

To determine how the activation of integrin αVβ3 at dendrites opens Cx43 HCs on the osteocyte cell body, we explored the downstream signals activated by αVβ3. Mechanical loading via liquid dropping at the dendritic processes (left panel) of MLO-Y4 cells opened HCs, as indicated by EtBr uptake (right panel) (Fig. 2a). Interestingly, the same cells that showed α5β1 integrin activation also exhibited HC opening; therefore, the integrin α5 activation on the cell body induced by the mechanical loading of dendrites correlated directly with HC activity (Fig. 2a). These data indicated the possible involvement of integrin α5β1 in the cell body. Activation of the α5 integrin, through its interaction with Cx43, opens HCs in response to mechanical stimulation.^22^ Inhibition of PI3K by LY294002 (LY), an upstream activating kinase of AKT, significantly blunted the activation of integrin α5 and HC opening induced by integrin αV activation at dendritic processes (Fig. 2a). We then asked whether the activation of intracellular PI3K signaling was sufficient to open HCs. Treatment with IGF-1, a known activator of PI3K signaling, activated AKT, and increased the expression of phosphorylated Akt473; however, LY significantly blocked this activation (Fig. 2b). MLO-Y4 cells were pretreated in the absence or presence of LY or an HC-blocking Cx43(E2) antibody and then incubated with IGF-1. IGF-1 induced HC opening as indicated by EtBr dye uptake, and this HC opening was decreased to basal levels by complete inhibition induced by the Cx43(E2) antibody (Fig. 2c). The PI3K inhibitor LY completely blocked the HC opening induced by IGF-1, suggesting that PI3K activation is responsible for HC opening.

**Fig. 3:**
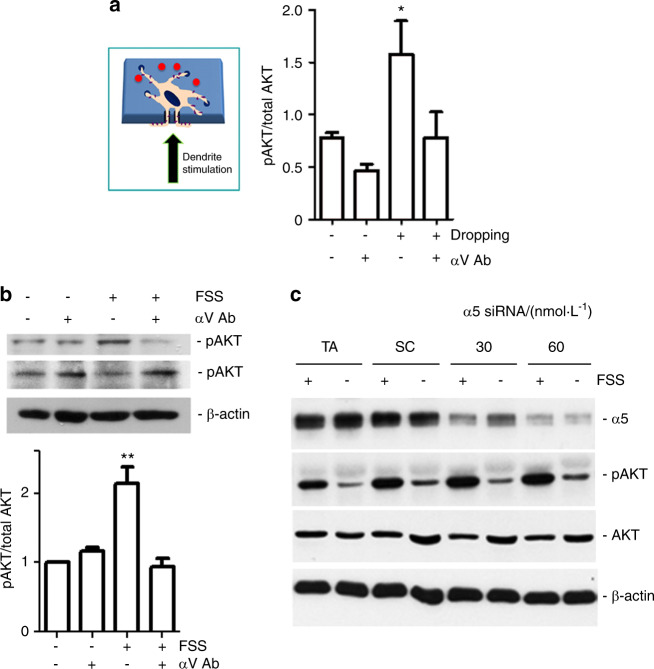
Integrin αV is required for PI3K-AKT activation induced by mechanical stress. **a** Inhibition of integrin αV prevents AKT activation induced by mechanical dropping on dendritic processes. MLO-Y4 cells cultured on transwell filter inserts were preincubated with an antibody blocking αV activation (αV Ab) for 20 min and mechanically stimulated by solution dropping on the dendritic process side of the transwell filter. The fixed cells were immunolabeled with an anti-phospho-AKT (Ser473) (pAKT) or total AKT antibody. The fluorescence intensities of pAKT and total AKT were quantified, and their ratio was calculated. Cells stimulated by dropping only *versus* all other treatments, *, *P* < 0.05. **b** Inhibition of integrin αV prevents AKT activation induced by FSS. MLO-Y4 cells were subjected to FSS for 30 min, and cell lysates were immunoblotted with an anti-pAKT or total AKT antibody (upper panel). The intensities of the protein bands were quantified, and the ratio was calculated and normalized to resting conditions (lower panel). Cells stimulated by FSS only *versus* all other treatments, **, *P* < 0.01. The data are presented as the mean ± SEM. *n* = 3. **c** Knocking down integrin α5 has no effect on AKT activation induced by FSS. MLO-Y4 cells were pretreated with 30 or 60 nmol·L^−1^ α5 siRNA, scrambled RNA (SC) or vehicle control (TA) for 48 h and then subjected to FSS for 30 min. Cell lysates were immunoblotted with an anti-α5, pAKT, AKT, or β-actin antibody

To determine whether PI3K signaling relies on integrin αVβ3 activation induced by mechanical stimulation, we applied mechanical stress, either through liquid dropping on a transwell filter (Fig. 3a) or FSS (Fig. 3b), to MLO-Y4 cells. Activation of PI3K-AKT signaling was determined by the ratio of phosphorylated AKT to total AKT (pAKT/total AKT). Increased AKT activation by mechanical loading was significantly inhibited by the integrin αV blocking antibody (αV Ab).

To further explore the possible involvement of integrin α5β1, AKT activation was assessed in cells treated with 30 or 60 nmol·L^−1^ siRNA targeting α5, 60 nmol·L^−1^ scrambled (SC) RNA, or vehicle (TA) (Fig. 3c). Integrin α5 siRNA at both concentrations dramatically decreased α5 expression; however, this knockdown had minimal impact on AKT activation induced by FSS. Together, these results suggest that the mechanical stress-induced activation of PI3K-AKT is mediated by integrin αV located at the dendritic processes of osteocytes, and this activation leads to HC opening.

### Integrin α5β1 on the cell body mediates HC opening by mechanical signals transmitted from osteocyte dendritic processes

**Fig. 4:**
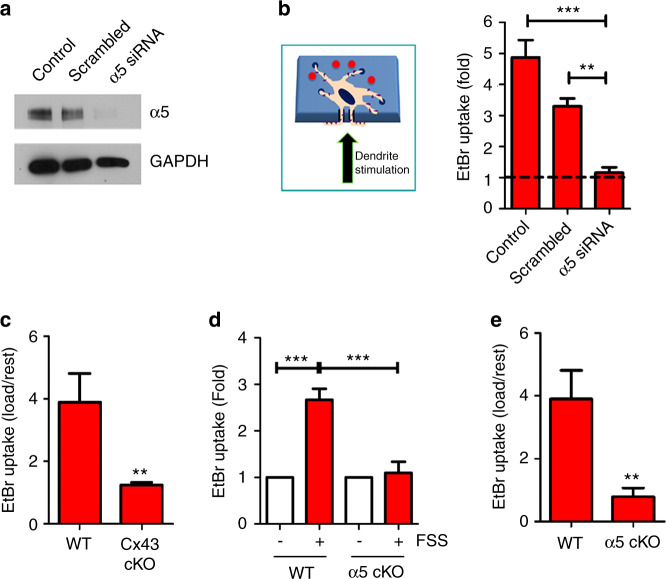
Mechanical stimuli at osteocyte dendritic processes are transmitted and open HCs through integrin α5. **a** Knockdown of integrin α5 expression by siRNA. MLO-Y4 cells were treated with α5 siRNA, scrambled RNA, or vehicle control for 48 h. Cell lysates were immunoblotted with an anti-α5 or GAPDH antibody. **b** Integrin α5 siRNA inhibits HC opening induced by mechanical loading on dendritic processes. Cells pretreated with α5 siRNA, scrambled RNA or a nontreatment control were mechanically stimulated by dropping 50 μmol·L^−1^ EtBr solution on the dendritic process (bottom) side of the filter (left panel). The extent of EtBr uptake was quantified on the cell body side of the transwell filter. The basal uptake is indicated by the dashed line. α5 siRNA versus the control and scrambled RNA-treated group. All data are presented as the mean ± SEM. *n* = 3. ***, *P* < 0.001, and **, *P* < 0.01. **c** Deficiency of osteocytic Cx43 in cKO mice attenuated the opening of Cx43 HCs by mechanical loading in situ. Tibial compression was conducted in WT and Cx43 cKO mice, and Evans blue was intravenously (IV) injected. After 2 h, the mice were perfused with PBS, and bone tissues were isolated. Evans blue uptake was evaluated by fluorescence signals, and the intensity of dye uptake in osteocytes was quantified by ImageJ. The data are presented as the mean ± SEM. *n* = 3. **, *P* < 0.01. **d** Deficiency of integrin α5 in osteocytes isolated from integrin α5 cKO mice attenuated the activation of Cx43 HCs by FSS. Primary osteocytes were isolated from 3-week-old WT and cKO mice and subjected to FSS for 15 min. EtBr uptake was conducted and quantified. The data are presented as the mean ± SEM. *n* = 3. ***, *P* < 0.001**. e** Deficiency of osteocytic integrin α5 in cKO mice attenuated the activation of Cx43 HCs by mechanical loading in situ. Tibial compression was conducted in WT and integrin α5 cKO mice, and Evans blue was IV injected. Evans blue uptake was evaluated by fluorescence (left panel), and the intensity of dye uptake in osteocytes was quantified by ImageJ (right panel). The data are presented as the mean ± SEM. *n* = 3. **, *P* < 0.01

Our previous studies showed that phosphorylation by AKT is essential for integrin interaction with Cx43 and HC opening.^23^ To determine whether integrin α5β1 is a receiver for signals transmitted by the dendritic process, we targeted α5 with siRNA (Fig. 4a, left panel). In the transwell assay in which dendritic processes were separated from the cell bodies, mechanical loading via liquid dropping at dendritic processes failed to open HCs in integrin α5 knockdown cells but not in vehicle (control) or scrambled RNA-treated control cells (Fig. 4b, right panel).

To assess HC opening and the regulatory effects of α5 integrin in vivo, we generated osteocyte-specific conditional KO (cKO) mice deficient for Cx43 or integrin α5 by crossing Cx43 or α5 flox/flox mice with mice in which Cre was driven by a 10-kb osteocyte-specific DMP1 promoter.^34^ DMP-1 is a relatively weak promoter that drives the expression of Cre. To circumvent this issue, an flx^+/-^ line is typically generated by crossing an flx/flx line with a knockout line. The western blot results showed comparable knockdown of integrin α5 in both Cre^+^;α5^flx/flx^ and Cre^+^;α5^flx/−^ mice (Fig. S2). HC activation analysis of Cre^+^;α5^flx/−^ mice was conducted using a tibial compression mouse model followed by the Evans blue dye uptake assay. Tibial loading opened HCs in osteocytes of wild-type (WT) mice; however, this activation was attenuated in Cx43-deficient osteocytes in situ (Fig. 4c). Primary osteocytes isolated from α5-deficient cKO mice impaired HC activation in response to FSS (Fig. 4d). Tibial loading data showed that similar to that in Cx43 cKO osteocytes, the opening of HCs was significantly ablated in integrin α5-deficient osteocytes in situ compared to the WT control (Fig. 4e).

### The dendritic integrin αVβ3 is more mechanosensitive than α5β1 in activating HCs by mechanical stress

**Fig. 5:**
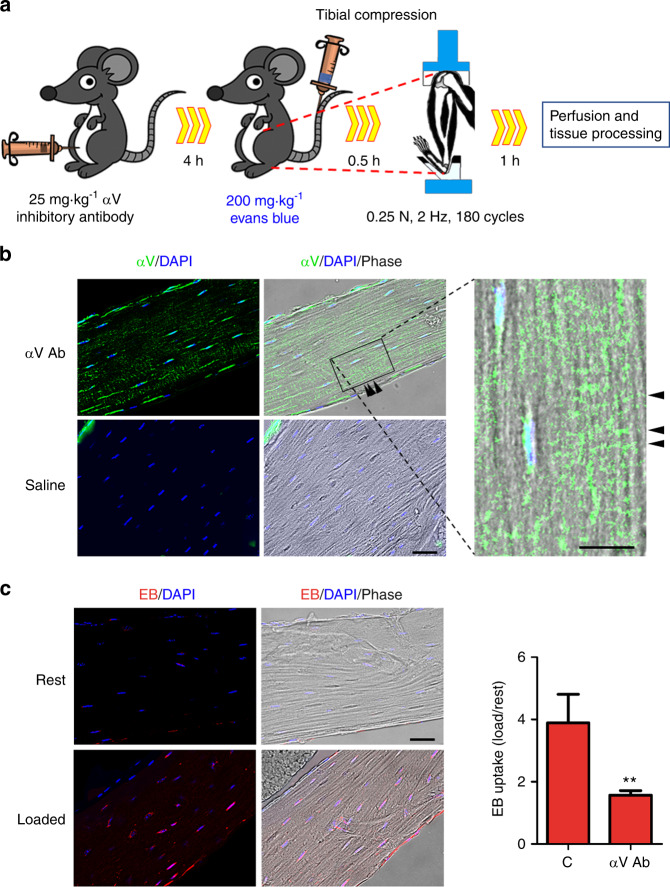
Integrin αV activation is required for osteocyte HC opening in vivo. **a** Schematic model of tibial compression in mice treated with an anti-αV inhibitory antibody. The antibody was IP injected 4 h before Evans Blue was IV injected. Tibial compression was conducted in WT mice injected with or without an antibody. **b** The anti-αV inhibitory antibody distribution in the bone was traced using Alexa 488-conjugated anti-Armenian hamster IgG. Bar, 50 µm. The 4.5X amplification of the image shows the reactivity distribution striated around the osteocyte dendrites (right panel, arrowheads). Bar, 25 µm. **c** Inhibition of osteocyte HC opening by an anti-αV inhibitory antibody. Evans blue uptake was evaluated by fluorescence signals (left panel). Bar, 50 µm. The intensity of dye uptake in osteocytes was quantified by ImageJ (right panel). The data are presented as the mean ± SEM. *n* = 3. **, *P* < 0.01

To elucidate the role of αVβ3 activation, we injected the αV inhibitory antibody into WT mice and applied tibial loading (Fig. 5a). Unlike the tibial bone loading protocol to determine anabolic function, tibial bone loading differed here in that the bone was mechanically loaded only once, and no woven bone was detected. Woven bone formation was not detected even under the identical loading magnitude for 10 days (Fig. S3a and b). After 10 days of tibial loading, the anabolic response was evident, with a significant increase in bone mineral density (BMD) being observed in WT mice (Fig. S3C). The distribution of the antibody signals appeared striated around the osteocyte lacuna, suggesting canaliculi distribution (Fig. 5b, image amplification in the right panel, arrowheads), and this observation was consistent with the presence of αV in osteocyte dendrites. As expected, tibial compression increased Evans blue uptake when compared with that of the contralateral leg (Fig. 5c, left panel). Application of the αV inhibitory antibody significantly inhibited the HC opening induced by tibial compression (Fig. 5c, right panel).

**Fig. 6:**
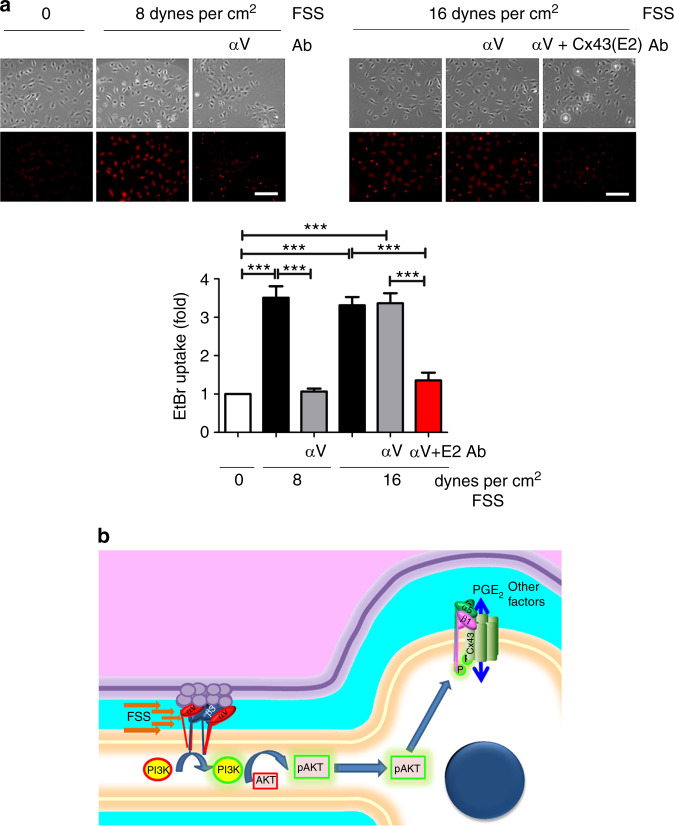
Integrin αV activation is required for HC opening at low but not high FSS levels. **a** Inhibition of integrin αV activation prevents HC opening at low but not high FSS levels. MLO-Y4 cells were pretreated with an antibody blocking αV activation (αV Ab) or an HC-blocking Cx43 (E2) antibody and then subjected to treatment with 8 dynes per cm^2^ FSS (upper, left panel), 16 dynes per cm^2^ FSS (upper, right panel), left untreated, or treated with a static control; EtBr dye was added. The degree of dye uptake was quantified (lower panel). Bar, 100 µm. The data are presented as the mean ± SEM. *n* = 3. ***, *P* < 0.001. **b** Mechanotransduction occurs in osteocytes through the functional interplay of integrins and Cx43 HCs. Integrin αVβ3 located at the dendrites of osteocytes as part of tethering elements facilitates the attachment of osteocytes to the canalicular wall, and this unique arrangement amplifies the magnitude of FSS experienced by osteocytes.^8,26^ FSS through the lacunae-canaliculi network induces the activation of αVβ3, which leads to the activation of intracellular PI3K and subsequent AKT activation. The activation of AKT results in the conformational activation of integrin α5β1. The phosphorylation of Cx43 by AKT and direct interaction between Cx43 and α5β1 open HCs,^23^ which allows the release of small molecule bone factors, such as PGE_2_, which are important for bone formation and remodeling

We designed an experiment to observe differences in the mechanosensitivities of αVβ3 and α5β1 to activate HCs. FSS at 8 dynes per cm^2^ induced HC opening, as indicated by EtBr uptake, and this opening was blocked by the αV inhibitory antibody (αV Ab) (Fig. 6a, upper left panel). However, the HC opening induced by 16 dynes per cm^2^ FSS was not inhibited by the αV antibody (Fig. 6a, upper right panel). The specificity of Cx43 HC activity was validated using an HC-blocking Cx43(E2) antibody, and the extent of dye uptake was quantified (Fig. 6a, lower panel). These data suggest that the mechanotransduction pathway initiated by αVβ3 at the dendrites can be activated at low FSS levels, which fail to activate integrin α5 directly on the cell body. However, a high FSS level directly activates α5, which may bypass the intracellular transmission pathway to open HCs. Due to differences in their sensitivities to mechanical stimulation, integrin molecules at different locations within osteocytes act coordinately to regulate biological functions in response to mechanical stresses of differing magnitudes. Together, our data from in vitro cell studies and in vivo mouse transgenic models with tibial loading showed that the activation of dendritic αVβ3 by mechanical loading transmits the signals to α5β1 on the cell body, leading to HC opening.

## Discussion

Osteocytes with extensive, long dendritic processes and a fluid-filled lacunae-canaliculi system are well positioned in the bone to sense mechanical stress and play a critical role in skeletal adaptation and anabolic responses to loading.^8^ In this study, we show that integrin αVβ3, located at the dendritic processes of osteocytes, serves as a distal mechanical sensor of osteocytes. As illustrated in Fig. 6b, activation of integrin αV transmits signals to the cell body to open HCs. This intracellular mechanotransduction pathway is mediated through the activation of intracellular PI3K-AKT signaling. The transmission of the signals to the cell body activates integrin α5β1 and consequently opens Cx43 HCs. We previously showed that α5β1 on the cell body regulates HC opening through its phosphorylation by AKT and direct interaction with Cx43.^22^ For the first time, we herein identify an intracellular mechanotransmission mechanism, initiating from mechanosensitive dendritic integrin molecules, that leads to the activation of intracellular signaling molecules and integrin molecules on the cell body, ultimately resulting in HC activation. We demonstrated this mechanotransduction pathway utilizing both cellular techniques in vitro and transgenic and mechanical loading models in vivo.

Osteocytes are highly responsive to dynamic or steady forms of fluid FSS, and the αVβ3 and α5β1 integrins in osteocytes can be activated after both types of mechanical stimulation.^22,27,35,36^ While different types of FSS exist, we used steady fluid flow in this study; osteocytes are reportedly more responsive to steady fluid flow than to oscillatory fluid flow, as indicated by the induction of more intracellular Ca^2+^ peaks with higher magnitudes.^37^ In our previous study, we compared steady and pulsatile flows and showed that their effects on Cx43 gap junction channels and HCs were comparable.^19,38^ Mechanical loading at physiological levels is shown to decrease sclerostin levels and promote anabolic function in IDG-SW3 osteocytes.^39^ We herein show that the inhibition of Cx43 HC activation with the Cx43(E2) antibody attenuated the SOST reduction in response to fluid shear stress, implicating the anabolic effect of functional Cx43 HCs during mechanical loading. We previously showed that Cx43 HCs activated by FSS serve as portals for the release of PGE_2_,^14^ which has been shown to function in an autocrine fashion to inhibit SOST expression.^40^ Therefore, decreased SOST expression likely results from the autocrine function of PGE_2_ or other factors being released through the opening of Cx43 HCs.

Interestingly, although high levels of mechanical loading can directly activate integrin α5 and open HCs, as shown in the current study with FSS as well as in previous studies on liquid dropping,^21^ this activation appears to originate primarily from integrin αV at dendritic processes under low FSS conditions. Compared to integrin α5, dendritic integrin αV is a more sensitive mechanosensor that can be activated by a lower level of mechanical stress on the cell body. This finding may be attributed to the fact that integrin αV is part of a “tethering” element, which attaches the dendritic processes of osteocytes to the canaliculi wall. According to the strain amplification model proposed by Weinbaum and colleagues,^8,25,26,28^ the force experienced at the “tethering” site is of a higher magnitude than that experienced without attachment. Because of this strain magnification, a low level of mechanical stimulation can be readily sensed and amplified by integrin αV. Therefore, mechanical signals must be transmitted by intracellular signals from dendritic processes to the cell body to activate integrin α5 and open HCs. Conversely, integrin α5 on the cell body can be directly activated by higher levels of mechanical stimulation. Unlike the dendritic integrin αV, the association of integrin α5 with its substrate, fibronectin, is not essential for HC opening, as we showed in our previous study.^22^ This evidence implies that an intracellular, not an extracellular, signal is responsible for the activation of integrin α5 and consequently for the opening of Cx43 HCs under low mechanical load conditions.

PI3K is activated in response to mechanical loading in osteocytes,^22^ and integrin αV is reported to activate PI3K by binding to focal adhesion kinase.^41^ Since the dendritic integrin αVβ3 is an initial mechanosensor in osteocytes, we investigated whether PI3K activation depends upon this integrin. Our data showed that PI3K was activated upon mechanical loading and that this activation could be blocked by integrin αV inactivation, suggesting that the activation of intracellular PI3K signaling relies on αV activation in response to mechanical loading. Very interestingly, we showed that IGF-1 also opened HCs, and this effect was mediated by the activation of PI3K signaling given that the inhibition of PI3K completely abolished the effect of IGF-1 on HC opening. These results support the notion that PI3K activation is sufficient to open HCs regardless of whether the signals are initiated by mechanical loading or other factors, such as IGF-1.

Knocking down integrin α5 does not affect PI3K, indicating that contrary to αV, PI3K signaling appears to be an upstream responsive element that bears the force-induced signal and transmits it to integrin α5β1, leading to its conformational activation. AKT is activated by PI3K signaling and is used as an indicator of PI3K activation. AKT has also been shown to be activated in response to mechanical loading in osteocytes.^42,43^ Our recent studies showed that FSS-activated AKT directly phosphorylates Cx43 and integrin α5 and that the phosphorylation of Cx43, in particular, plays a predominant role in its interaction with α5;^23^ this interaction on the cell body is essential for HC opening.^23^ Therefore, AKT is likely the effector of activated signaling that regulates HC opening, and HC opening leads to the release of molecules that promote bone cell responses to loading, resulting in an anabolic environment.

In addition to osteocyte cell models with mechanical loading, either with FSS or liquid dropping, we validated a mechanotransduction mechanism in vivo using two osteocyte-specific mouse cKO models with mechanical stimulation via axial compression on tibias. Tibial compression opened HCs in only WT osteocytes and not in those of Cx43 cKO mice with osteocyte-specific Cx43 deficiency, which suggests that the Cx43 HCs in osteocytes are responsive to mechanical loading. We also showed the impairment of HCs in α5-deficient osteocytes, which occurred for both isolated primary osteocytes and osteocytes in situ. Interestingly, when WT mice were injected with an αV inhibitory antibody and subjected to tibial loading, the inhibition of HCs in osteocytes was significantly but incompletely inhibited compared to that of the untreated control. This result may have been due to the partial effectiveness of the antibody. Alternatively, it may also be explained by the aforementioned mechanism related to the differences between αVβ3 on dendrites and α5β1 on the cell body. High levels of mechanical loading may, at some threshold, directly activate α5β1, which can then bypass the requirement for activation through the αVβ3 pathway.

This study focused on the mechanism of mechanotransduction from osteocyte dendritic processes to the cell body via the interplay of two types of integrins and Cx43 HCs. Previous studies have reported the results of Cx43 knockout mouse models. When Cx43 was deleted in mature osteoblasts, endocortical bone formation was attenuated in response to four-point tibial bending.^44^ In addition, in three other studies, osteoblast precursors, mature osteoblasts, and osteocytes with Cx43 deletion all showed enhanced periosteal formation,^45–47^ and one study showed attenuated endocortical formation.^48^ In Cx43 knockout models, both gap junctions and HCs are compromised, not just HCs, and gap junctions reportedly play significantly different roles from HCs, with HCs being highly responsive to mechanical loading in osteocytes. Furthermore, whether other proteins and pathways are upregulated to potentially compensate for the deletion of Cx43 remains unknown, and a compensatory mechanism has been reported in other gene knockout models. Other channels are also reported to be involved in the mechanotransduction of osteocytes, such as the P2X7-pannexin1 channel and the recently identified Piezo1 channel.^49–51^

We found that upon exposure to mechanical loading of various magnitudes, osteocytes responded differently, and this difference could be partially explained by the sensitivity of mechanical sensors and integrins on the osteocyte cell surface. Our data showed that low levels of mechanical stress, which were insufficient to activate α5β1 integrins, activated αvβ3 integrins located in dendrites. Due to the unique anatomical structure of dendritic processes, the shear stress level is substantially magnified, possibly via the “tethering element”, which attaches osteocyte dendrites to the canaliculi wall. A higher level of shear stress, however, could directly activate α5β1 on the osteocyte cell body and open HCs. Unlike osteocytes in vivo, no canaliculi are present, and the binding of integrin αvβ3 to the matrix can therefore amplify the mechanical stimulation. We previously showed that in contrast to αvβ3 activation, that of α5β1 in osteocytes is independent of its association with the extracellular matrix.^22^ Based on this evidence, osteocytes, integrins, and HCs appear to behave similarly in situ and in cell culture in response to mechanical loading. In our previous studies, we applied FSS and fluid dropping to osteocytes and found that integrins and HCs, as well as PI3K/AKT signaling, were similarly activated by these two forms of mechanical loading.^21,22^ Nevertheless, because of technical limitations, we cannot exclude other differences induced by different forms of mechanical stimulation. Some study limitations include the impact of matrix properties, duration, and frequency of mechanical stimulation. To mitigate the variation between cellular environments in vitro, we used a transwell system that allows separation of the osteocyte cell body and dendritic processes. The fluid dropping approach permitted us to specifically apply mechanical stimulation to the dendritic processes of osteocytes.^21^ Our data suggest that PI3K/AKT is a major signaling pathway by which Cx43 HCs are opened, and inhibition of this pathway attenuates the effect of integrins on Cx43 HC activation. However, we cannot exclude the involvement of other signaling pathway(s), especially those activated by mechanical loading in bone remodeling. It is well acknowledged that crosstalk exists between most major signal transduction pathways,^52^ with examples including the PI3K/AKT, Ca^2+^, and PKA pathways, all of which are activated by mechanical loading.

Together, the results of this study establish the critical link between intracellular mechanotransduction pathways and their effects on the anabolic activity of bone through the functional interplay of integrin subtypes, cell signaling molecules, and connexin channels. Elucidation of the functional interplay between integrins and connexin channels will substantially advance mechanobiology in general. Moreover, this discovery may lead to therapeutics involving the specific targeting of HCs for bone loss due to not only bone diseases such as osteoporosis but also aging and space flight or immobilization, which result in a lack of mechanical loading.

## Materials and methods

### Cell culture and reagents

MLO-Y4 osteocytic cells derived from murine long bones were cultured on rat tail collagen type I-coated surfaces and grown in α-modified essential medium (α-MEM) supplemented with 2.5% fetal bovine serum (FBS) and 2.5% bovine calf serum (BCS) as described previously.^53^ Preosteocyte IDG-SW3 cells were cultured in α-MEM supplemented with 5 ng·mL^−1^ IFNγ at 5% CO_2_ and 33 °C. After reaching confluence, they were cultured in differentiation medium (α-MEM supplemented with 10% FBS, 1% penicillin-streptomycin, 50 μg·mL^−1^ ascorbic acid and 4 mmol·L^−1^ β-glycerophosphate) for 9 days to promote differentiation into osteocytes.^32^ MLO-Y4 cells were also plated on rehydrated (250 μL of media for 30 min) transwell filter inserts (Biocoat^®^ Cell Culture Inserts, 1.0 μm pores, BD Biosciences, Bedford, MA). The transwell occupancy rate was ~80%–85%, and the cells were ~50% confluent. The cells were then either subjected to mechanical stimulation or processed for immunostaining. The Cx43(E2) antibody was generated and affinity purified as previously described.^19^ An antibody targeting integrin α5 (CD49e) (R&D Systems, MN), a mouse anti-β3 antibody (Developmental Studies Hybridoma Bank, Iowa City, IA), integrin αV clone H9.2B8 (BD Bioscience, NJ), and tripeptide RGD (Biomol, PA) were used in this study. WOW1 was generously provided by Dr. Sanford Shattil (University of California at San Diego), and a glutathione S-transferase (GST)-tagged portion of fibronectin protein 9–11 (GST-FNIII_9-11_) was generously provided by Dr. Martin Schwartz (Yale University).

### Isolation of primary osteocytes from long bone tissues of α5 cKO mice

The protocol for preparing primary osteocytes from bone pieces was modified from a previously published method.^54^ Briefly, long bones from 3- to 4-week-old mice were dissected, and soft tissues and bone marrow were removed. The bones were cut into pieces ~2 mm in length and digested by alternate usage of collagenase type I and ethylenediaminetetraacetic acid (EDTA) on a rotating shaker in a CO_2_ incubator at 37 °C. After multiple treatments with collagenase type I and EDTA to remove other bone cells, the bone pieces were plated on a collagen-coated dish in α-MEM supplemented with 2.5% FBS and 2.5% BCS and left untouched for 9 days. The osteocytes from the bone pieces were removed by trypsinization and seeded on collagen-coated glass to apply FSS in α-MEM supplemented with 2.5% FBS and 2.5% BCS.

### Immunofluorescence labeling, integrin activation assay, and immunoblotting

For total cell labeling, the cells were fixed in 4% paraformaldehyde (PFA) for 10 min at room temperature (RT), blocked and permeabilized with 2% goat serum, 2% fish skin gelatin, 0.025% Triton X-100, and 1% bovine serum albumin in phosphate-buffered saline (PBS). The cells were incubated overnight at 4 °C with affinity-purified antibodies against Cx43(CT) (1:300 dilution) and integrin β3 (1:500 dilution) and then with the appropriate secondary antibody for 1 h. For cell surface labeling, cultured cells were washed with PBS and then incubated with cold α-MEM supplemented with 2.5% FBS, 2.5% BCS, 10 mmol·L^−1^ HEPES, and an integrin αV inhibitory antibody (1:500 dilution) at 4 °C for 1 h. The cells were rinsed 3 times with PBS, fixed in 4% PFA, blocked and permeabilized, and then labeled with secondary antibodies in succession for 1 h each at RT. Slides were mounted using Vectashield Mounting Medium (H-1000, Vector Laboratories), and confocal fluorescence imaging was performed using a confocal laser scanning microscope (Fluoview; Olympus Optical, Tokyo, Japan).

MLO-Y4 cells cultured for 48 h were subjected to FSS, and integrin activation assays were performed according to a previously published method.^29^ Briefly, FSS-treated cells were incubated with antibodies targeting GST-FNIII_9-11_ (20 μg·mL^−1^), WOW-1 (1:100 dilution), or inactive αV for 30 min at 37 °C. Next, the cells were fixed with 2% PFA and then incubated with secondary antibodies propagated in goats, including Alexa488-conjugated anti-mouse immunoglobulin G (IgG) for WOW1, Alexa 488-conjugated anti-Armenian hamster IgG for the αV inhibitory antibody, rhodamine-conjugated anti-mouse IgG for integrin β3, and rhodamine-conjugated anti-rabbit IgG for Cx43. To assess the involvement of PI3K signaling, cells were pretreated with the PI3K inhibitor LY294002 for 30 min prior to FSS and then incubated with GST-FNIII_9-11_, WOW-1, or inactive integrin αV as described above.

For immunoblotting assays, cultured MLO-Y4 cells were lysed in lysis buffer (5 mmol·L^−1^ Tris, 5 mmol·L^−1^ EDTA/ethylene glycol tetraacetic acid, pH 8.0), and cell lysates were immunoblotted with an anti-AKT (1:1 000 dilution), anti-phospho-AKT (1:1 000 dilution), anti-integrin α5 (1:1 000 dilution), anti-SOST (1:1 000 dilution), anti-β-actin (1:500 dilution), or anti-glyceraldehyde 3-phosphate dehydrogenase (1:5 000 dilution) antibody. Crude membrane extracts were prepared by centrifugation at 100 000 × *g* for 30 min, immunoblotted with an antibody and detected by corresponding secondary antibodies and enhanced chemiluminescence (Amersham Pharmacia Biotech, Piscataway, NJ).

### FSS and a mechanical loading assay on transwell filters

FSS was created by parallel plate flow chambers separated by a gasket of defined thickness with gravity-driven fluid flow using a peristaltic pump as previously described.^38^ The thickness of the gasket determined the channel height. By adjusting the channel height and flow rate, stress levels of 8 and 16 dynes per cm^2^ were generated. Controls consisted of MLO-Y4 cells in S-minimum essential medium (SMEM) not subjected to FSS, and the circulating medium was SMEM. The entire flow system was encased within a CO_2_ incubator at 5% CO_2_ and 37 °C.

The mechanical stimulation of MLO-Y4 or differentiated IDG-SW3 cells in transwell inserts was conducted by solution dropping as described previously.^21^ Briefly, 50 μL of SMEM was passed through a pipette from a height of 5.7 cm in the center and at three edges of the membrane (four drops total per well) from either side of the filter. The filters were then placed on their side in a clean well with 500 μL of either SMEM or the dye solution, 50 μmol·L^−1^ EtBr. After washing with PBS, the cells were fixed with 2% PFA. The transwell membrane was then peeled from the inserts and mounted onto glass slides using Vectashield Mounting Medium (H-1000, Vector Laboratories). Images were taken with a Zeiss Epifluorescence microscope using the appropriate filters.

### Integrin α5 siRNA treatment

MLO-Y4 cells were trypsinized, resuspended in antibiotic-free OPTI medium (Invitrogen, Carlsbad, CA), and then transiently transfected with integrin α5 siRNA or scrambled siRNA (Ambion, Austin, TX) using a siRNA transfection kit (Ambion). The cells were harvested at 48 h after transfection and assessed for the expression of integrin α5 and β-actin or HC activity utilizing dye uptake.

### Generation of osteocyte-specific Cx43 and α5 conditional knockout mice and in vivo injection of integrin antibodies

We generated Cx43 osteocyte-specific conditional knockout (cKO) mice (DMP1-Cre; Cx43^fl/-^) as previously described.^55^ Mice with heterozygous ITGA5 (α5^+/-^) gene deletion and the floxed ITGA5 gene (α5^flx/flx^) were generated and generously provided by the laboratory of Dr. Richard Hynes.^56^ Mice with an osteocyte deletion of the α5 integrin were generated using the Cre/Lox system. First, mice with a floxed ITGA5 gene (α5^flx/flx^) were crossed with α5 heterozygous mice expressing one ITGA5 allele (α5^+/−^). We then crossed mice expressing a Cre recombinase driven by a 10-kb DMP1 promoter, which leads to gene expression predominantly in osteocytes,^34^ (DMP1-Cre) with α5^flx/−^ mice to generate α5 osteocyte-specific conditional knockout mice (DMP1-Cre; Cx^flx/−^) or (DMP1-Cre; α5^flx/−^). Genotyping was performed by polymerase chain reaction techniques using genomic DNA isolated from mouse tails and corresponding primers synthesized at the UTHSCSA DNA Core Facility.

### In vivo tibial compression model

We subjected 4-month-old WT and Cx43 transgenic mice to mechanical loading through tibial compression^57–61^ using a loading setup established in our laboratory. For WT mice, we also intraperitoneally (IP) injected the αV inhibitory antibody (H9.2B8) at a concentration of 25 mg·kg^−1^. Mice were subjected to tibial loading at a frequency of 2 Hz using a haversine waveform for 600 cycles with a constant force (8.86 N for control mice and 7.44 N for Cre- α5^flx/null^ mice) for 5 min. We applied dynamic peak loads to achieve 1 200 με.

### In vitro and in vivo dye uptake assays

MLO-Y4 cells, differentiated IDG-SW3 cells, and primary osteocytes isolated from WT and transgenic mice were subjected to FSS for 10 min, liquid dropping, or treatment with 5 ng·mL^−1^ insulin-like growth factor 1 (IGF-1) for 30 min. Dye uptake experiments were performed as described previously.^14^ Briefly, the cells were incubated with 50 μmol·L^−1^ EtBr dye mixture for 5 min, and the mean intensity of the nuclear region was determined using ImageJ software (NIH). Cells cultured for 48 h were preincubated with the antibody blocking integrin αV activation (0.5 ng·mL^−1^) or the Cx43(E2) antibody (2 μg·mL^−1^) for 30 min. To provide accurate assessments of HC activity among different assays, we presented dye uptake amounts as fold changes over the basal controls.

In vivo dye uptake assays were conducted with 4-month-old WT, Cx43, and α5 cKO mice; a second group of WT mice was also treated with the αV inhibitory antibody. Mouse tibial compressions were performed on the left leg, and the right leg was used as an unloaded control. The αV inhibitory antibody (25 mg·kg^−1^) was IP injected 4 h before Evans blue injection. Mice were intravenously (IV) injected with Evans blue dye (200 mg·kg^−1^) via the tail vein. Thirty minutes after IV injection, tibial compression was performed, and 1 h later, the mice were sacrificed with isoflurane and perfused with PBS through the left ventricle of the heart. The volume of PBS used for perfusion was ~3 times the blood volume to remove the excess dye in the blood. Then, the animals were perfused with 4% PFA (3 times the blood volume) to fix the tissues, followed by perfusion with PBS to remove excess PFA in the mouse body system. Tibial bones were collected, decalcified with 10% EDTA (pH 7.4) for 2 weeks, embedded in optimum cutting temperature compound, and frozen in liquid nitrogen for cryostat tissue sectioning. Frozen tissue sections of 12 μm thickness were prepared and counterstained with 4′,6-diamidino-2-phenylindole (DAPI, 1 μg·mL^−1^ for 5 min) before imaging on a fluorescence microscope. Evans blue dye uptake in the cortical bone area was analyzed by measuring the fluorescence intensities of 20 cells per section (3 sections per mouse), and images were quantified by ImageJ software.

### Statistical analysis

All data were analyzed using GraphPad Prism 5.04 software (GraphPad). One-way ANOVA and the Student-Newman Keul’s test were used for comparisons of two or more groups, and the unpaired Student’s t-test was used for comparisons between two groups. Unless otherwise specified in the figure legends, the data are presented as the means ± SEMs of at least three experiments. An asterisks indicate the degree of significance compared with the controls (*, *P* < 0.05, **, *P* < 0.01, ***, *P* < 0.001).

## Supplementary information

Text

Text with marked up

Figures
