# Supplementary material for: Mechanotransduction via the coordinated actions of integrins, PI3K signaling and Connexin hemichannels
Source: Bone Res. 2021 Feb 2;9:8. doi: 10.1038/s41413-020-00126-w (PMC7854719; doi:10.1038/s41413-020-00126-w)
Supplement: Supplementary file 1 — Text [file 41413_2020_126_MOESM1_ESM.docx]

Supplemental figures:

**Figure S1. Cx43 is expressed in differentiated osteocytes and are sensitive to mechanical stimulation.** IDG-SW3 cell line that replicates osteoblast to osteocyte differentiation is indicated by the expression of GFP driven by an osteocyte marker DMP1 promoter. **(A)** Cx43 signals (red) detected by anti-Cx43 antibody and GFP (green) increase after 9 days in differentiation media compared with control. The nuclei were stained with DAPI (blue). Bar, 50 µm. **(B)** Mechanical loading through dye dropping (DP) induces ethidium bromide (EtBr) uptake in 9 days differentiated IDG-SW3 cells and this this uptake was inhibited by a specific Cx43 hemichannel blocking Cx43E2 antibody (E2). Ethidium bromide fluorescence intensity was quantified (right panel). Data are presented as mean ± SEM. *, P<0.05.; **, P<0.01. Bar, 100 µm.

**Figure S2. Reduction of integrin α5 expression in cKO mice.** DMP1-promoter driven cre conditional integrin α5 KO mice were produced by crossing DMP1-cre^+/-^ mice with α5^flx/-^ mice. Proteins were isolated from the cortical bones (around middle shaft region of femur) of adult DMP1-cre^-^;α5^flox/-^, DMP1-cre^+^;α5^flx/flx^, and DMP1-cre^+^;α5^flx/-^ mice. Fifty micrograms of proteins were separated on SDS-PAGE and immunoblotted with anti-α5 or anti-GAPDH antibody. Conditional KO with DMP1 promoter driven Cre decreased α5 expression in cortical bones of DMP1-cre^+^;α5^flx/flx^ and DMP1-cre^+^;α5^flx/-^ mice (Lanes 3 and 4).

**Figure S3. Anabolic effects of mechanical loading on WT mice.** 4-month-old WT mice were subjected to tibial loading at a frequency of 2 Hz using a Haversine waveform for 600 cycles with a constant force of 8.86N 0.25 N during the dwell period every day for 10 days with a two-day break in between. We applied dynamic peak loads to achieve 1200 με. (A) Representative coronal view images from microcomputed tomography (μCT) scanning of tibia of WT control and tibia loaded groups after 10 days of mechanical loading. The bone regions highlighted with light blue rectangles were magnified as indicated (blue arrows). (B) Representative three-dimensional model of tibia mid-shaft cortical region of WT control and loaded groups. (C) Bone mineral density (BMD) was measured by DEXA analysis before and after 10 days of loading. The percentage of BMD change was calculated. Data shown are mean ± SEM. *, P < 0.05. n = 8.
