# Supplementary figures and images for: Mechanotransduction via the coordinated actions of integrins, PI3K signaling and Connexin hemichannels
Source: Bone Res. 2021 Feb 2;9:8. doi: 10.1038/s41413-020-00126-w (PMC7854719; doi:10.1038/s41413-020-00126-w)

Fig. S1

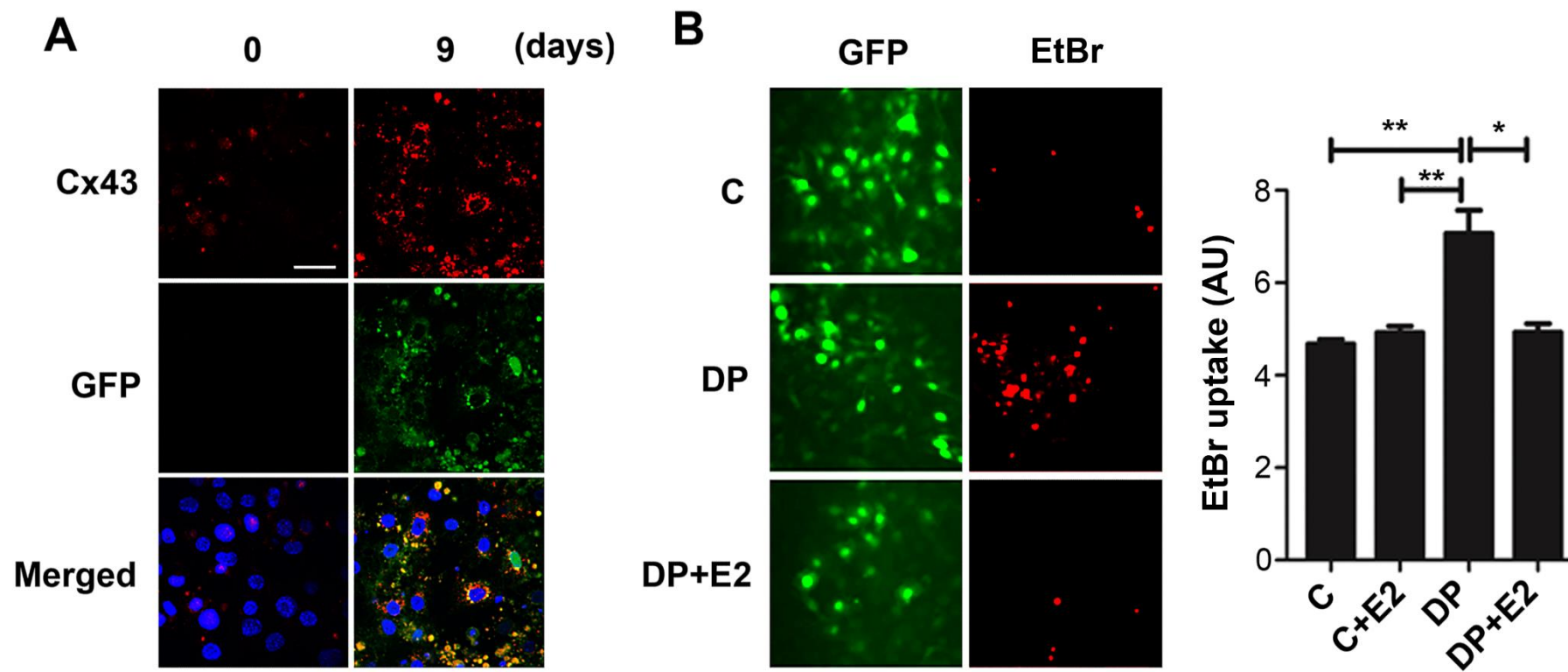

Fig. S2

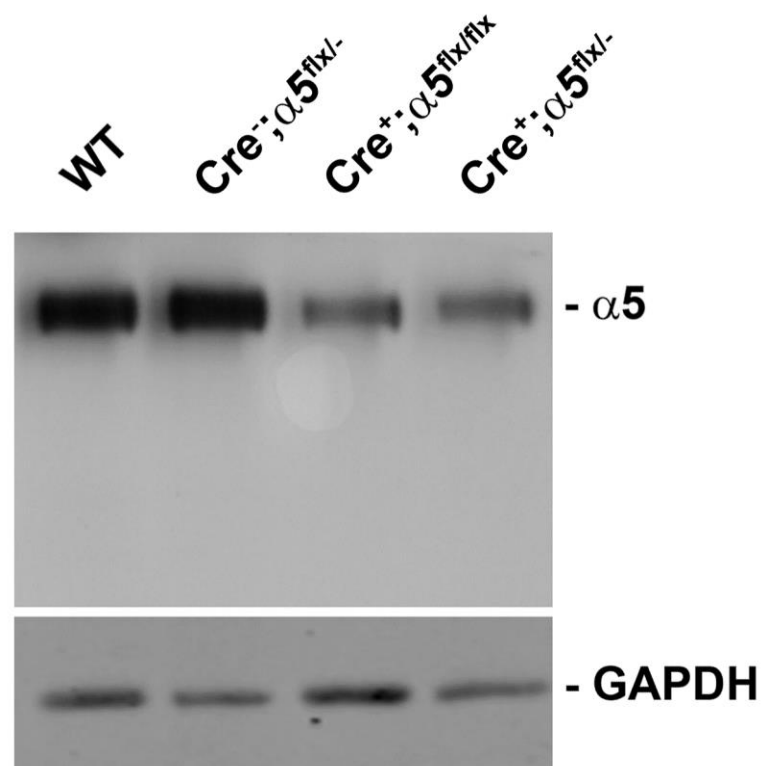

Fig. S3

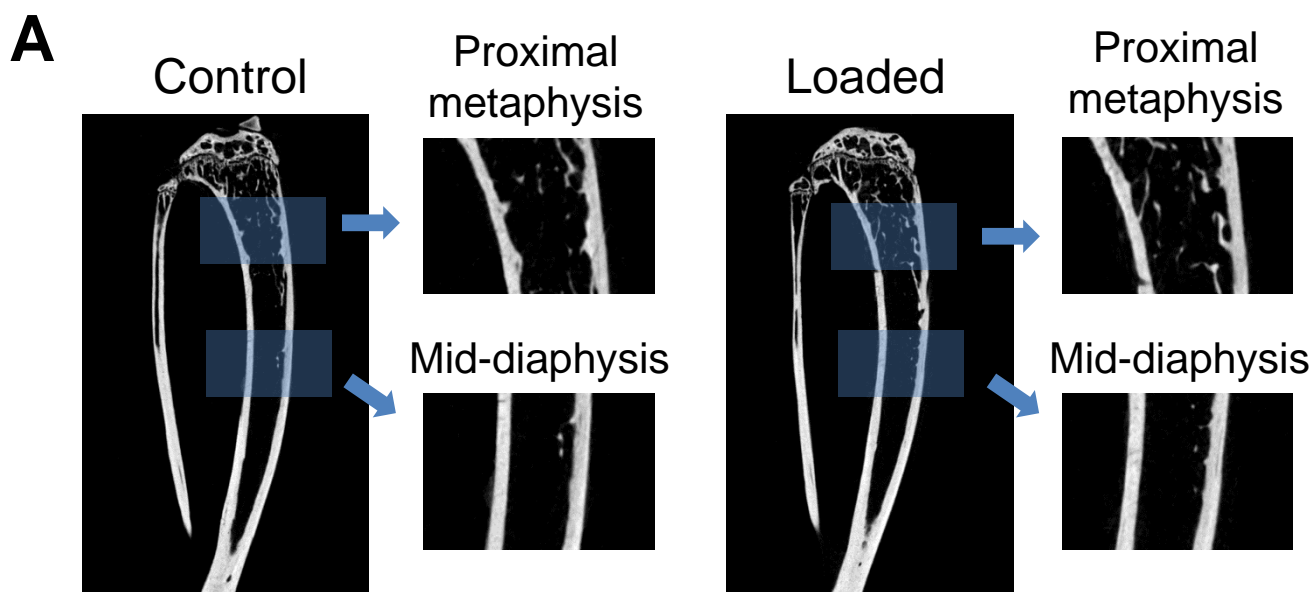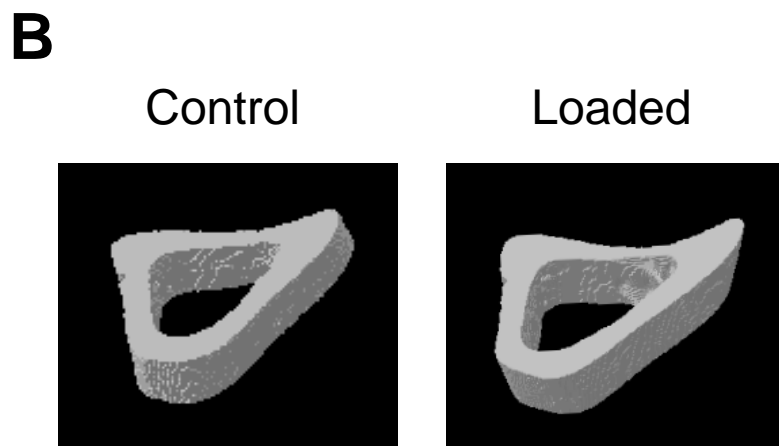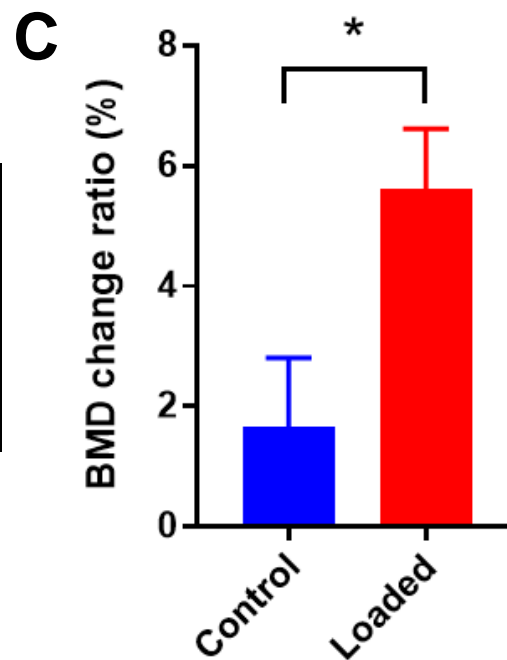

Supplement: Supplementary file 3 — Figures [file 41413_2020_126_MOESM3_ESM.pdf]
